# Reliability of miRNA Analysis from Fixed and Paraffin-Embedded Tissues

**DOI:** 10.3390/ijms20194819

**Published:** 2019-09-27

**Authors:** Eros Azzalini, Eleonora De Martino, Paolo Fattorini, Vincenzo Canzonieri, Giorgio Stanta, Serena Bonin

**Affiliations:** 1 DSM-Department of Medical Sciences, University of Trieste, 34149 Trieste, Italy; azzalinieros@yahoo.it (E.A.); edemartino@units.it (E.D.M.); fattorin@units.it (P.F.); vcanzonieri@cro.it (V.C.); stanta@impactsnetwork.eu (G.S.); 2 Doctorate of Nanotechnology, University of Trieste, 34100 Trieste, Italy; 3 Centro di Riferimento Oncologico di Aviano (CRO) IRCCS, 33081 Aviano, Italy

**Keywords:** miRNA, Bouin’s, formalin, ddPCR, real-time PCR

## Abstract

In clinical practice, patients’ tissues are fixed and paraffin-embedded in order to enable histological diagnosis. Nowadays, those tissues are also used for molecular characterization. Formalin is the most used fixative worldwide, and Bouin’s solution in some worldwide institutions. Among molecular targets, micro RNAs (miRNAs), the single-stranded non-coding RNAs comprised of 18 to 24 nucleotides, have been demonstrated to be resistant to fixation and paraffin-embedding processes, with consequent possible application in clinical practice. In the present study, *let-7e-5p*, *miR-423-3p*, *miR-92a-1-5p*, *miR-30d-5p*, *miR-155-5p*, *miR-200a-3p*, and *miR-429* were investigated in formalin and matched Bouin’s solution-fixed tissues of high grade serous ovarian cancers by means of real-time and droplet digital PCR (ddPCR). Micro RNAs were detectable and analyzable in both formalin- and Bouin’s-fixed specimens, but on average, higher Ct values and lower copies/µL were found in Bouin’s-fixed samples. Data from formalin-fixed samples correlated significantly for most targets with Bouin’s ones, except for *let-7e-5p* and *miR-155-5p*. This study shows that miRNAs are analyzable in both formalin- and Bouin’s-fixed specimens, with the possibility, after proper data normalization, to compare miRNA-based data from formalin-fixed samples to those of Bouin’s-fixed ones.

## 1. Introduction

Tissue fixation is the primary step for pathological analysis to prevent the autolysis of tissues and allow further histological examination. For this purpose, in diagnostics, chemical fixatives are mainly used, and among them the most widely used is formalin, the aqueous solution of formaldehyde. However, Bouin’s fixative has been extensively used as an elective fixative for histological examination in some institutions worldwide [1]. Bouin’s fixative is a mixture of a saturated aqueous solution of picric acid, formalin, and acetic acid [2]. The principle of fixation is mostly based on protein precipitation through picrate formation. This fixation method has been used for specific purposes because it better preserves some morphological details, such as nuclear conformation [3]. In the past decades, several studies have already shown that Bouin’s-fixed samples are amenable for nucleic acid and protein analyses, but only to a certain point, as the fixative has resulted to be more detrimental compared to 10% buffered formalin mixture in the preservation of biomolecules [1,3,4]. Currently, no investigation has been carried out on the analysis performance with respect to miRNAs, despite the publication of two samples in a report [4]. 

Among biomolecules, micro RNAs (miRNAs), which are single-stranded non-coding RNAs comprised of 18 to 24 nucleotides, have been launched as a new generation of biomarkers because of their possible use in monitoring the efficacy, as well as the safety, of therapeutic regimens, but also in the diagnosis or risk assessment for the development of a disease, as well as for treatment options [5]. Furthermore, they have been demonstrated to be resistant to formalin-fixed paraffin-embedding (FFPE) processing [6] and storage [7] in archives so that they can be analyzed in retrospective studies. 

The aim of this study was to investigate the reliability of micro RNA analysis in formalin- and Bouin’s-fixed tissue by analyzing 15 matched samples of formalin- and Bouin’s-fixed paraffin-embedded high grade serous ovarian cancers (HGSOC) as part of the HERCULES project [8].

## 2. Results

Micro RNA analyses included in this study were performed on 15 matched cases of high grade serous ovarian carcinomas (collected between 2002 and 2009), where formalin- and Bouin’s solution-fixed blocks were available. 

### 2.1. Yield and Purity 

**Figure 1:**
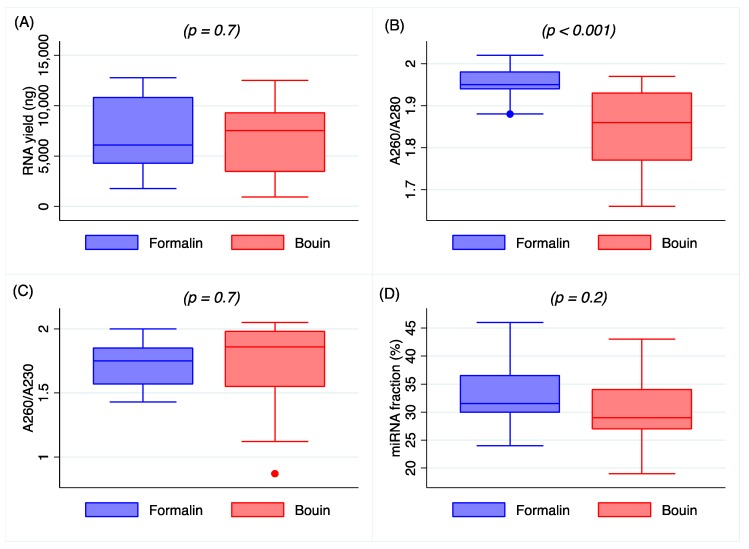
Box plot representing total RNA yield (**A**), RNA purity by A260/280 ratio (**B**), by A260/230 ratio (**C**), and isolated miRNA fraction (**D**) in matched formalin- and Bouin’s-fixed samples.

RNA yield was comparable between FFPE and Bouin’s-fixed samples, as shown in Figure 1A (*p* = 0.7). Mean yield for formalin was 7.04 µg (range 1.778–12.76 µg), while for Bouin’s-fixed samples was 6.63 µg (range 0.93–12.52 µg). Overall, A260/280 ratio was on average higher than the 1.8 threshold in all samples, although pairwise testing revealed that phenol/protein contamination as shown by A260/280 ratio resulted significantly higher in Bouin’s-fixed samples when compared to FFPE ones (*p* < 0.001), as shown in Figure 1B. A260/230 ratio was comparable between formalin- and Bouin’s-fixed specimens (*p* = 0.7, Figure 1C) with a mean value of 1.7, which is slightly lower than the optimal threshold, possibly indicating chaotropic salt contamination due to the isolation procedure.

### 2.2. Integrity 

Ten nanograms of total RNA from each sample was submitted to Agilent 2100 Bioanalyzer (Agilent Technologies, Santa Clara, CA 95051, USA). The assay allowed investigating the distribution of small RNA fragments quantifying the smallest RNA fraction, which corresponds to microRNAs in the range size from 10 to 40 nt. The miRNA content was measured as the relative abundance in comparison to the total small RNA fraction. In all samples, BioAnalyzer electropherograms showed a characteristic profile with the presence of a unique distinct peak in the range of 10–40 nt. Furthermore, a mean fraction abundance of 33% (range: 24–46%, median 31.5%, SD 5.7) was obtained from formalin and 31% (range: 19–43%, median 29%, S.D. 6.0) from Bouin’s-fixed samples without any difference between the two fixatives (*p* = 0.2), as shown in Figure 1D. 

**Figure A1:**
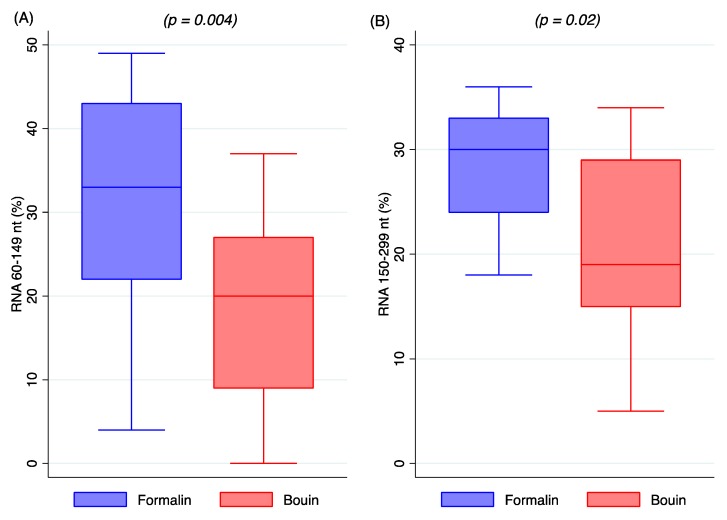
Box plot representing the relative amount of RNA stretches of (**A**) 60–149 nucleotides and (**B**) 150–299 nucleotides in matched formalin- and Bouin’s solution-fixed samples. The box represents the first and third quartiles, intersected by the median, and the whiskers are the range.

Total RNA analysis by Agilent Bioanalyser (see the original outputs in the Appendix B) returned a median RIN of 2.4 in formalin- and of 2.5 in Bouin’s-fixed samples without any significant difference (*p* = 0.3). However, the relative amount of RNA stretches of 60–149 nt and 150–299 nt resulted significantly higher in formalin (mean value 31% and 28%) than matched Bouin’s samples (mean value 19% for 60–149 nt and 20% for 150-299 nt) (*p* = 0.04 and *p* = 0.02, respectively), as shown in Figure A1 in Appendix A.

### 2.3. qRT-PCR 

Data on real-time PCR efficiencies detected in formalin- and Bouin’s-fixed specimens are reported for each miRNA analyzed in Table 1.

In the present study, two reference miRNAs were selected, *let-7e-5p* and *miR-423-3p*, because of their stability in high grade serous ovarian cancer, while *miR-92a-1-5p*, *miR-30d-5p*, *miR-155-5p*, *miR-200a-3p*, and *miR-429* were chosen for their content in guanine-cytosine (GC) and their expression in ovarian cancers [9,10,11,12]. Furthermore, *miR-92a-1-5p* was selected for its lower expression level in ovarian cancer [9]. Therefore, the aforementioned miRNA levels were analyzed in matched pairs of formalin- and Bouin’s-fixed paraffin-embedded tissues. 

**Figure 2:**
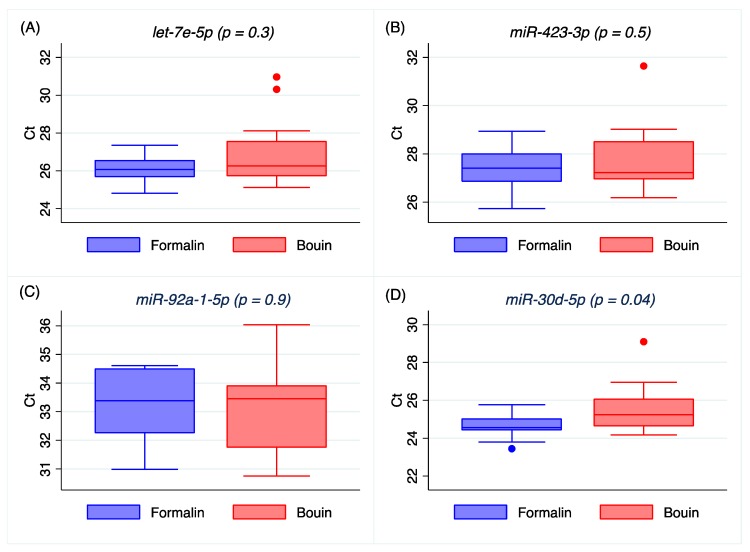
Box plot representing quantitative reverse transcription polymerase chain reaction (RT/qPCR) of *let-7e-5p* (**A**), *miR-423-3p* (**B**), *miR-92a-1-5p* (**C**), and *miR-30d-5p* (**D**) in matched formalin- and Bouin’s-fixed tissues by real-time PCR. The box represents the first and third quartiles, intersected by the median, and the whiskers are the range.

**Figure A2:**
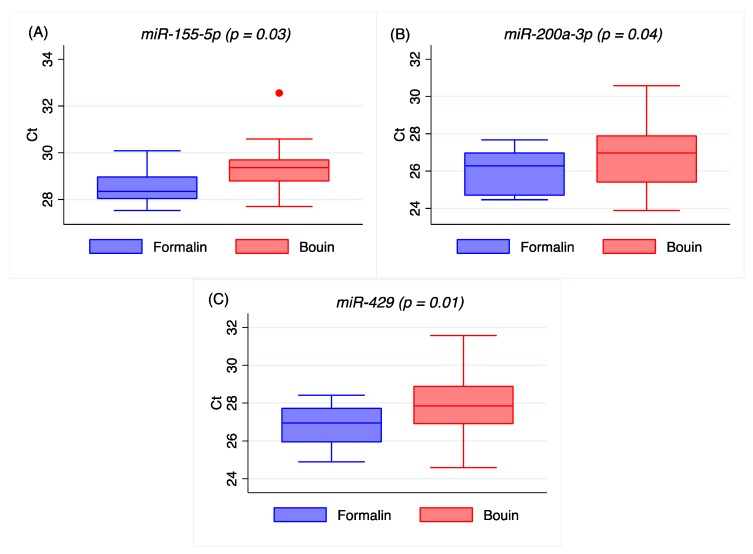
Box plot representing quantitative reverse transcription polymerase chain reaction (RT/qPCR) of *miR-155-5p* (**A**), *miR-200a-3p* (**B**), and *miR-429* (**C**) in matched formalin- and Bouin’s-fixed tissues by real-time PCR. The box represents the first and third quartiles, intersected by the median, and the whiskers are the range.

Real-time PCR was successful in all samples for all miRNAs analyzed. The expressions of *let-7e-5p*, *miR-423-3p*, and *miR-92a-1-5p* were closely comparable in FFPE and matched Bouin’s specimens (*p* = 0.3, *p* = 0.5, and *p* = 0.9, respectively), as shown in Figure 2A–C, but it was significantly different for *miR-30d-5p* (*p* = 0.04, Figure 2D), *miR-155-5p* (*p* = 0.03, Appendix A, Figure A2A), *miR-200a-3p* (*p* = 0.04, Appendix A, Figure A2B), and *miR-429* (*p* = 0.01, Appendix A, Figure A2C), which resulted to be detectable at significant higher Ct values in Bouin’s-fixed samples. Median Ct values and coefficients of variation for the analyzed miRNAs are reported in Table 2. Overall, Ct values detected in Bouin’s fixatives resulted to have a higher coefficient of variation compared to formalin for each analyzed miRNA. 

Pairwise Spearman’s rank analysis showed a significant correlation between formalin and Bouin’s specimens for *miR-423-3p* (*p* = 0.003), *miR-92a-1-5p* (*p* = 0.0002), *miR-30d-5p* (*p* = 0.01), *miR-200a-3p* (*p* = 0.005), and *miR-429* (*p* = 0.004), but not for *let-7e-5p* (*p* = 0.8) or *miR-155-5p* (*p* = 0.4), as reported in Table 3. By comparing Ct values in formalin- and Bouin’s-fixed specimens, a significant correlation was found by regression analysis for *miR-423-3p* (*p* = 0.03), *miR-92a-1-5p* (*p* = 0.0003), *miR-30d-5p* (*p* = 0.02), *miR-200a-3p* (*p* = 0.004), and *miR-429* (*p* = 0.008), but not for *let-7e-5p* (*p* = 0.4) or *miR-155-5p* (*p* = 0.8), as shown in Table 3. This result shows that the assessment of *let-7e-5p* and *miR- 155-5p* is influenced by the fixation processes in formalin and Bouin’s solution, likely due to the different degradation and chemical modification extent of the two fixation methods. 

### 2.4. Droplet Digital PCR

**Figure 3:**
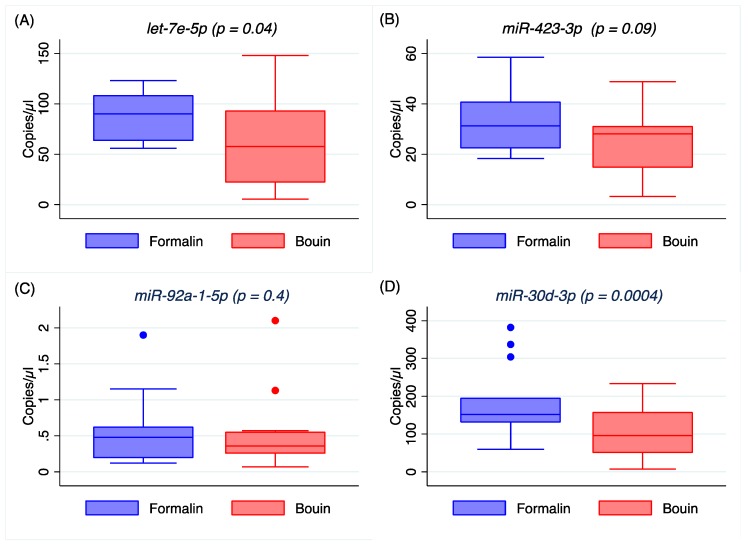
Box plot representing the quantitative reverse transcription polymerase chain reaction (RT/ddPCR) of *let-7e-5p* (**A**), *miR-423-3p* (**B**), *miR-92a-1-5p* (**C**), and *miR-30d-5p* (**D**) in matched formalin- and Bouin’s-fixed tissues by ddPCR. The box represents the first and third quartiles, intersected by the median, and the whiskers are the range.

**Figure A3:**
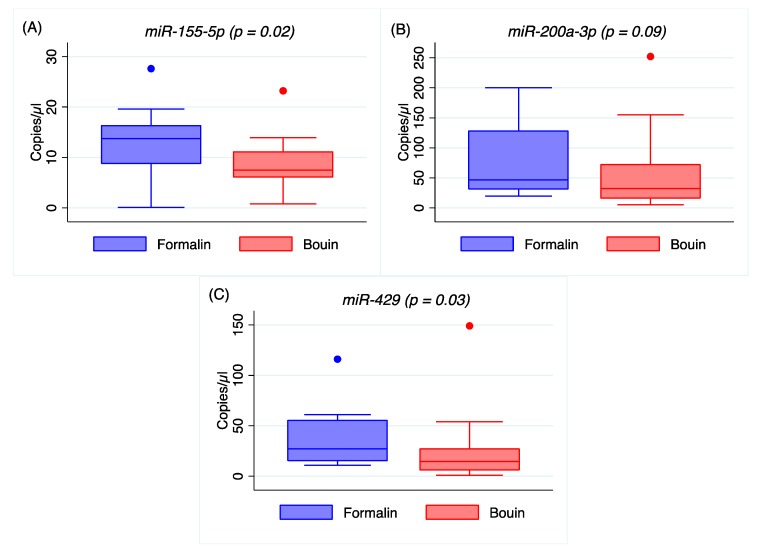
Box plot representing quantitative reverse transcription polymerase chain reaction (RT/ddPCR) of *miR-155-5p* (**A**), *miR-200a-3p* (**B**), and *miR-429* (**C**) in matched formalin- and Bouin’s-fixed tissues by ddPCR. The box represents the first and third quartiles, intersected by the median, and the whiskers are the range.

The expression of the seven miRNAs was further assessed by ddPCR. Comparing miRNA expression in formalin- and Bouin’s-fixed samples, we found a significant difference between the two groups for *let-7e-5p* (*p* = 0.04), *miR-30d-5p* (*p* = 0.0004), *miR-155-5p* (*p* = 0.02), and *miR-429* (*p* = 0.03), but not for *miR-423-3p* (*p* = 0.09), *miR-92a-1-5p* (*p* = 0.4), or *miR-200a-3p* (*p* = 0.09), as shown in Figure 3 and Figure A3 (Appendix A). 

Median copies/µL and coefficient of variations are reported in Table 2. For every miRNA, the median copies in formalin-fixed samples were higher than in Bouin’s ones, where the coefficient of variation was also higher. Pairwise Spearman’s rank analysis did not return any correlation between matched pairs for either *let-7e-5p*, *miR-423-3p*, or *miR-155-5p* (*p* = 0.4, *p* = 0.5, and *p* = 0.1, respectively), but it returned a significant correlation for *miR-92a-1-5p* (*p* = 0.007), *miR-30d-5p* (*p* = 0.005), *miR-200a-3p* (*p* = 0.008), and *miR-429* (*p* = 0.01), as reported in Table 3. This result has also been confirmed by linear regression, which resulted to be statistically significant for *miR-92a-1-5p* (*p* < 0.0001), *miR-30d-5p* (*p* = 0.0006), *miR-200a-3p* (*p* = 0.0001), and *miR-429* (*p* = 0.0005), but not for *let-7e-5p* (*p* = 0.5), *miR-423-3p* (*p* = 0.3), or *miR-155-5p* (*p* = 0.05) (Table 3). 

### 2.5. miRNA Comparison: qRT-PCR vs. ddPCR

**Figure 4:**
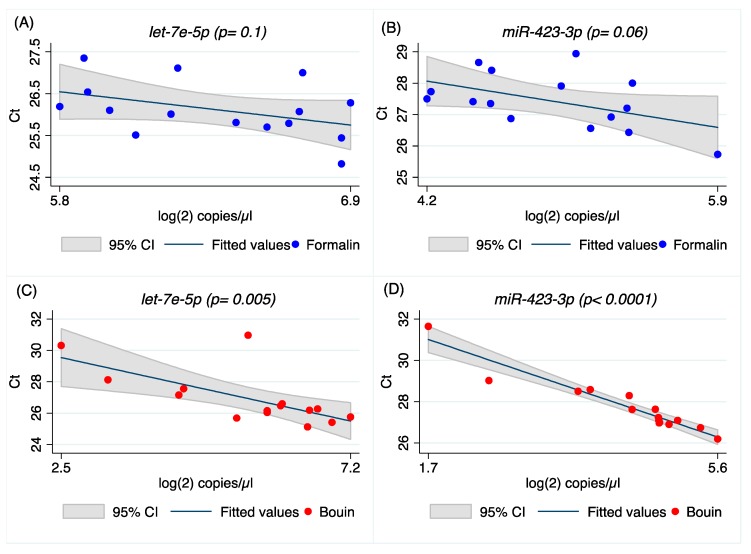
Scatter plot and linear prediction with 95% confidence interval of real-time and ddPCR results for *let-7e-5p* and *miR-423-3p* in formalin- (**A**,**B**) and Bouin’s-fixed tissues (**C**,**D**), respectively.

**Figure 5:**
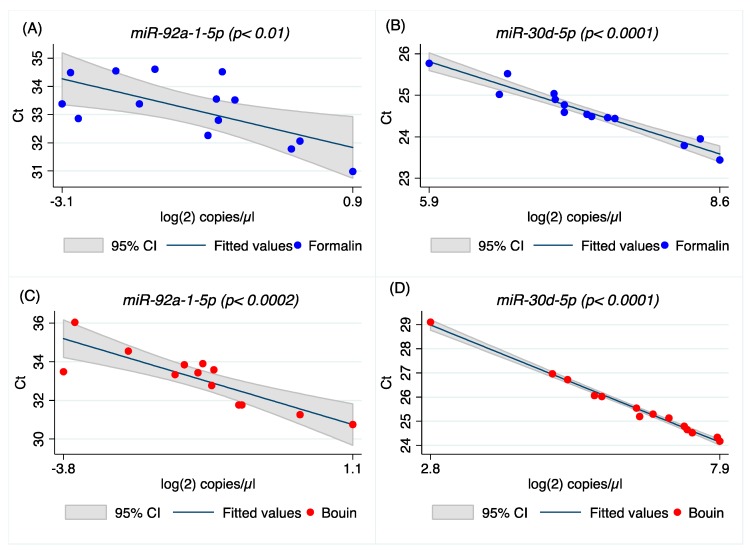
Scatter plot and linear prediction with 95% confidence interval of real-time and ddPCR results for *miR-92a-1-5p* and *miR-30d-5p* in formalin- (**A**,**B**) and Bouin’s-fixed tissues (**C**,**D**), respectively.

**Figure A4:**
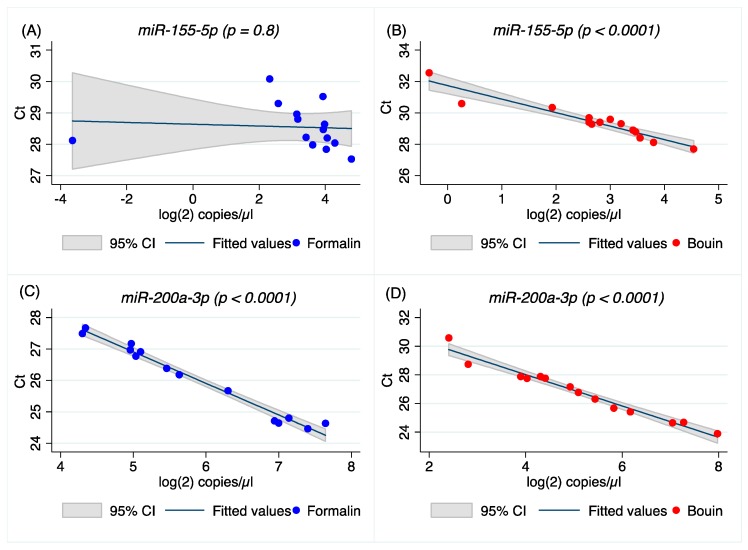
Scatter plot and linear prediction with 95% confidence interval of real-time and ddPCR results for *miR-155-5p* and *miR-200a-3p* in formalin- (**A**,**B**) and Bouin’s-fixed tissues (**C**,**D**), respectively.

**Figure A5:**
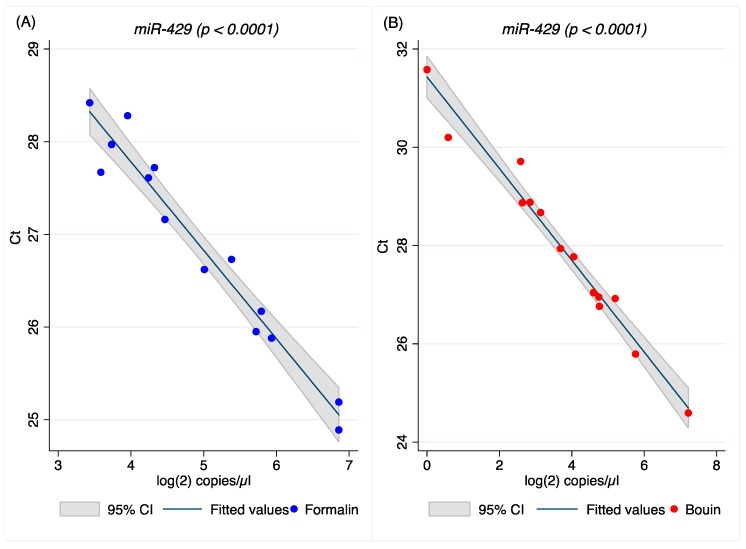
Scatter plot and linear prediction with 95% confidence interval of real-time and ddPCR results for *miR-429* in formalin- (**A**) and Bouin’s-fixed tissues (**B**), respectively.

In order to correlate qRT-PCR and ddPCR measurements, the same amount of cDNA (0.4 ng) was submitted to PCR in each assay on both platforms. The resulting data, expressed as cycle threshold (Ct) and the log(2) of the number of target copies/µL, were compared by linear regression model and Spearman’s correlation test for both formalin- and Bouin’s-fixed samples, as reported in Figure 4 and Figure 5, and in Appendix A Figure A4 and Figure A5. Our results clearly show that there is a linear correlation between ddPCR results and real-time PCR for both formalin- and Bouin’s-fixed samples as regards *miR-92a-1-5p*, *miR-30d-5p*, *miR-200a-3p*, and *miR-429*. For *let-7e-5p*, *miR-423-3p*, and *miR-155-5p*, real-time and ddPCR results were significantly correlated only in Bouin’s-fixed samples. Linear regression analysis produced an R-square (R^2^) value of 0.46 (*p* = 0.005) for *let-7e-5p*, 0.91 (*p* < 0.0001) for *miR-423-3p*, and 0.9 (*p* < 0.0001) for *miR-155-5p* in Bouin’s-fixed tissues (Figure 4C,D, Table 4, and Figure A4 and Figure A5 in Appendix A). 

Similarly, results from qPCR and ddPCR for *miR-92a-1-5p* were significantly correlated in both formalin- (*p* = 0.01) and Bouin’s-fixed tissues (*p* = 0.0002) (see Figure 5A,C and Table 4). These results were also confirmed for the other microRNAs analyzed in this study, as reported in Table 4, Figure 5, and in Appendix A Figure A4 and Figure A5.

### 2.6. qRT-PCR miRNAs Normalization 

**Figure 6:**
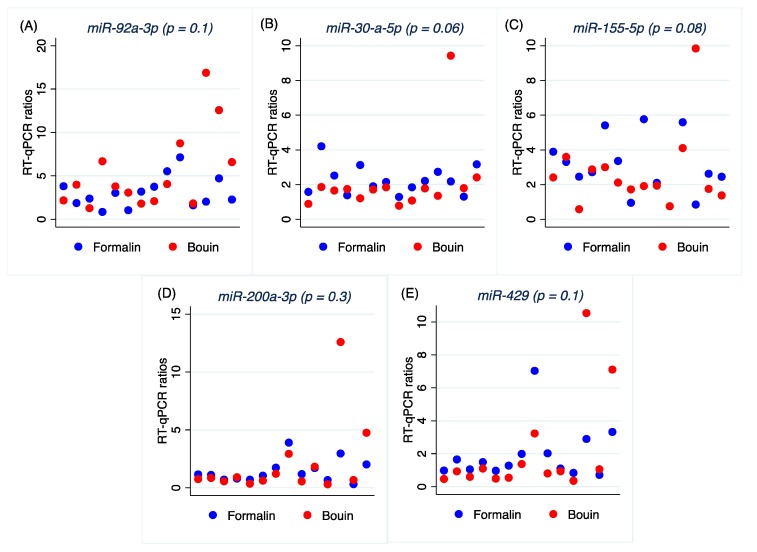
Scatter plot of real-time RT-qPCR ratios for *miR-92a-1-5p* (**A**), *miR-30d-5p* (**B**), *miR-155-5p* (**C**), *miR-200a-3p* (**D**), and *miR-429* (**E**) in formalin- and Bouin’s-fixed tissues.

In order to normalize qRT-PCR data, the geometric mean of the most stable miRNAs, namely *let-7e-5p* and *miR-423-3p*, was used as reference miRNA. Normalized qRT-PCRs were submitted to Wilcoxon’s matched-pairs signed-ranks test, which did not return any significant difference between formalin and Bouin’s ratios, as shown in Table 5 and Figure 6.

## 3. Discussion

In this study, we investigated whether miRNAs can be efficiently isolated and quantified from formalin- and Bouin’s-fixed paraffin-embedded tissues for expression analysis by real-time and droplet digital RT-PCR. For this purpose, we compared the results in matched Bouin’s- and formalin-fixed paraffin-embedded samples of high grade serous ovarian cancers. Degradation of nucleic acids in fixed tissues is due to different contributing factors related to enzyme activity, and also to the effect of chemicals. In detail, in formalin fixation, nucleic acid degradation assembles both fragmentation and chemical modification of methylol addition to the bases [13]. The latter factor is even more critical for RNA, as the methylol addition impedes reverse transcription and cDNA synthesis [13]. The fixatives analyzed in the present study include both a crosslinking agent, formaldehyde (formalin), but our results clearly show that their degradation effects on RNA during fixation greatly differ.

Data on the extraction procedure indicate that total RNA obtained from formalin and Bouin’s specimens was closely comparable in amount, although a higher purity was detected in formalin extracts, as shown in Figure 1B. The lower A260/A280 ratio in Bouin’s extracts is likely related to the presence of picric acid residues, that, with its aromatic ring, has an absorbance peak between 200 and 300 nm [14]. Nonetheless, the amount of small RNAs as detected by Agilent Bioanalyzer resulted as being similar in extracts from formalin- and Bouin’s-fixed samples, highlighting the feasibility of the use of these type of tissues. 

Micro RNAs, considering their short length, have been reported to be more accessible in FFPE tissues compared to miRNAs, representing a viable analysis for clinical research and diagnosis [15]. In several reports, miRNAs have been shown to be minimally affected by FFPE treatments, as expression levels of isolated miRNAs were directly comparable in frozen and FFPE tissue samples [16,17,18]. 

Our results on real-time PCR detection for *let-7e-5p*, *miR-423-3p*, and *miR-92a-1-5p* show similar Ct between matched formalin and Bouin’s specimens, but not for *miR-30d-5p*, *miR-155-5p*, *miR-200a-3p*, or *miR-429*, which were detectable at significantly higher Ct in Bouin’s fixative (Figure 2 and Figure A2 in Appendix A). In addition, the coefficients of variation of all the analyzed miRNA were lower in formalin- than in Bouin’s-fixed specimens. Using matched fixed samples of the same surgical tissue in this study, the higher Ct levels detected in some miRNAs in Bouin’s-fixed tissues are presumably due to the sample degradation level. Consequently, the trend of higher Ct values and CV detected in Bouin’s is in agreement with a lower amount of transcript in such samples, which is strictly linked to the RNA fragmentation as shown by the Agilent rRNA fragmentation analysis. Therefore, our results point out lower levels of degradation caused by lower fragmentation in formalin-fixed rather than in Bouin’s-fixed specimens. Moreover, the ddPCR assessment is in agreement with higher extent of RNA fragmentation in Bouin’s samples: These samples, indeed, had lower copies/µL than formalin for all miRNAs investigated. In particular, this trend is even more evident for *miR-30d-5p* because of its lower expression level in ovarian cancers. The highest fragmentation of the RNAs in Bouin’s fixative is in line with the fixative composition, which has a 9–10% concentration of formaldehyde, 5% glacial acetic acid, and 0.9% of picric acid [19], and it is in agreement also with results reported by other authors [3,4,20,21,22]. Only Gloghini and colleagues have demonstrated the detection of 921 base stretches by RT-PCR from both formalin- and Bouin’s-fixed tissues, but the sample fixation time was reduced to 5 hours [23].

Real-time PCR results were significantly correlated between Bouin’s- and formalin-fixed specimens for most miRNA analyzed, but not for *let-7e-5p* and *miR-155-5p*, as shown by Spearman’s and regression analyses. Although a possible explanation could be related to the different sequence of the miRNA in terms of GC content (*let-7e-5p* has 41% and *miR-155-5p* 38%, respectively) as it seems that miRNAs with GC% of less than 40% are significantly degenerated in FFPE specimens [24], data from *miR-200a-3p* and *miR-429* (GC content 41% and 36%, respectively) do not support this hypothesis. The absence of correlation between formalin- and Bouin’s-fixed tissues for *let-7e-5p* and *miR-155-5p* seems to support a different preservation of those two miRNAs in Bouin’s and formalin fixatives. However, we cannot exclude that this result is an artifact stemming from the customized design of PCR assays for those two miRNAs. Regarding the analyzed fixatives, both are formalin-based, however the contribution of the nitrogenous bases modification by those fixatives is virtually unknown. It is well known that adenine is the most modified nitrogenous base after formalin fixation [25], so it is reasonable to suppose that formalin fixation could alter the detectability of some miRNAs with higher adenine content. Given the results of rRNA on fragmentation showing a higher fragmentation in Bouin’s-fixed samples, it is reasonable to hypothesize that one possible difference between the two fixatives arises from a higher modification of RNA in formalin-fixed specimens. We acknowledge that Bouin’s fixative has a high percentage of formalin, but no data are available at present on the modification rate of adenine residues in Bouin’s-fixed nucleic acids. Given the composition of Bouin’s fixative, it is likely that the activity of formalin, including its ability to modify adenine residues by CH_2_OH addition, could be inhibited by the low pH due to the presence of picric acid [21]. In Bouin’s solution, indeed, the effects of formalin and picric and acetic acids balance each other: (i) Formalin fixes cytoplasm, hardens tissues, and prevents paraffin penetration; (ii) picric acid leaves tissue soft and coagulates cytoplasm, compensating for most the unduly effects of formalin; (iii) the tissue shrinking effect of picric acid is compensated by acetic acid [26].

A higher correlation between ddPCR and real-time PCR results was obtained for all miRNAs investigated in Bouin’s-fixed specimens, as shown in Figure 4, Figure 5, Figure A4 and Figure A5 (the latter two in Appendix A). In Bouin’s fixative, a lower deviation from the linear prediction and a tighter confidence interval were detected. One possible hypothesis to explain the lower correlation detected in FFPE samples could be related to a higher amount of modified bases, due to formaldehyde addition to the RNA bases that can act as a PCR inhibitor. This is evident when comparing real-time PCR results with ddPCR because the latter has already shown higher resilience to PCR inhibitors [27]. It was already demonstrated that DNA isolated from FFPE tissue itself exhibits an inhibitory effect on PCR, leading to unstable amplification [28]; therefore, for RNA, we hypothesize a similar behavior, presumably due to the adducts.

Bouin’s fixative consists of picric acid, acetic acid, and also formaldehyde, having both a coagulative as well as cross-linking effect on proteins [22], with a possible minor modification of nitrogenous bases due to the acidic pH. The possible inhibitor activity of the mono-methylol adducts in formalin extracts is supported also by the lower amplification efficiencies detected in formalin compared to Bouin’s (Table 1). Any deviation of the PCR efficiency from 1 can provide a measure of the level of PCR inhibition in the sample [29]. Nevertheless, we acknowledge that a limitation on the abovementioned hypothesis is the small amount of RNA processed to generate cDNA (20 ng) and the high dilution used for PCR analyses (40*X*). 

Our results show that by choosing the proper reference miRNAs with a calibrator that includes both formalin- and Bouin’s-fixed samples, it is possible to eliminate differences in the expression profiles of miRNAs and analyze in the same cohort samples processed with both fixatives. Although no significant differences were obtained in the expression profiles in formalin- and Bouin’s-fixed samples, we acknowledge that specific care should be taken for outliers and further validation studies are needed to verify that finding. 

In conclusion, our results indicate that microRNAs can be analyzed in formalin- and Bouin’s-fixed samples as well. Our data support for a higher fragmentation of miRNA from Bouin’s-fixed tissues, but a possible lower level of nitrogenous bases modification by formalin. Thus, miRNA expression studies can be reliably performed by real-time PCR or, better, by ddPCR in routinely obtained pathological material fixed in formalin or Bouin’s fluid, but considering proper data normalization correcting sample-to-sample degradation.

We acknowledge as limitations of this study that no analyses have been made in fresh frozen samples for a further validation of results, and that no analyses have been carried out to test the level of adenine modification in the analyzed specimens. As a future perspective, the chemical analysis of the RNA templates obtained from Bouin’s-fixed samples is planned.

## 4. Materials and Methods

### 4.1. Samples

A total of 30 paraffin-embedded tissue blocks from 15 patients were collected at the National Cancer Institute of Aviano. Informed consent was obtained from all individual participants included in the study. The study was conducted in accordance with the Declaration of Helsinki, and it was approved by the Institutional Review Board of CRO-Aviano (protocol number 1213, 24/01/2017). For each patient, two matched tissue blocks of the same surgical specimen were retrieved, one fixed in formalin and one in Bouin’s solution, for a total of 15 matched pairs. All cases were obtained from debulking surgeries of high grade serous ovarian cancers, carried out from 2002 to 2009, of pT3c grade and stage III. HGSOC cases have been selected for the HERCULES project funded by the European Union’s Horizon 2020 research and innovation program under grant agreement No 667403. Fixation and embedding procedures were those routinely performed in the laboratory at the time of patient’s surgery with a conventional fixation for 24 h.

### 4.2. miRNA Isolation from Formalin- and Bouin’s-Fixed Paraffin-Embedded Tissues

From each paraffin-embedded block, one 10-μm-thick section was cut and collected into 1.5-ml microcentrifuge sterile tubes. miRNA isolation was carried out by the use of the Maxwell RSC^®^ extractor (Promega, Madison, WI 53711-5399, USA) using a purification protocol which allows extracting miRNAs from fixed tissues as suggested by the manufacturer. In detail, tissue de-waxing was carried out by the use of 300 µL of mineral oil as provided by the Maxwell^®^ RSC RNA FFPE kit (Promega, Madison, WI 53711-5399, USA; code AS1440). Procedures of the abovementioned kit were strictly followed for protein digestion by proteinase K and the DNAse digestion step. Afterwards, the aqueous solution of digested samples was transferred into the cartridge of the Maxwell^®^ RSC miRNA tissue kit (Promega, Madison, WI 53711-5399, USA; code AS1460) to allow miRNA, as well as total RNA, isolation following the manufacturer’s procedures. Elution of the samples was done in 30 µL of nuclease-free water (Promega, Madison, WI 53711-5399, USA).

### 4.3. RNA Quantification and Quality

RNA concentration and purity were measured by Nanodrop ND 1000 spectrophotometer (Thermo Scientific, Waltham, MA 02451, USA) using 1 µL of isolated RNA. The A280/260 and A260/230 absorbance ratios were used to assess purity, considering a ratio between 1.8 and 2.0 to be pure.

RNA and miRNA integrity was estimated by microcapillary electrophoresis in an Agilent 2100 Bioanalyzer (Agilent Technology, Santa Clara, CA 95051, USA). For miRNA analysis, sample aliquots were diluted at 10 ng/µL just before use and measured in the Agilent 2100 Bioanalyzer using a Small RNA kit (Agilent Technology, Santa Clara, CA 95051, USA). The integrity of miRNA was calculated as the relative abundance of miRNA species (10–40 nt) in comparison to the total amount of small RNA fraction (10–150 nt). For RNA integrity, 1 µL of RNA was submitted to Agilent 2100 Bioanalyzer using Agilent RNA 6000 nano kit (Agilent Technology, Santa Clara, CA 95051, USA). RIN number and relative percentage of total RNA species (60–149 nt and 150–299 nt) were recorded.

### 4.4. cDNA Synthesis

Twenty nanograms of RNA was reverse transcribed into cDNA in 10 µL final volume, using the miRCURY LNA RT (Qiagen, Hilden, Germany) according to manufacturer’s instructions. The cDNA was then split into aliquots of 2 µL and stored at −80 °C until use. To cover the methods’ set-up and analysis, cDNA synthesis was made in duplicate with 20 ng of RNA each time.

### 4.5. Real-time PCR

miRNA *let-7e-5p* (MIMAT0000066), *miR-423-3p* (MIMAT0001340), *miR-92a-1-5p* (MIMAT0004507), *miR-30d-5p* (MIMAT0000245), *miR-155-5p* (MIMAT0000646), *miR-200a-3p* (MIMAT0000682), and *miR-429* (MIMAT0001536) were analyzed by real-time PCR. *miR-423-3p* and *let-7e-5p* were chosen as they have been reported to be stably expressed in high grade serous ovarian cancer [30]. The remaining ones were chosen for their content in GC and their expression in ovarian cancers [9,10,11,12]. Complementary DNA aliquots were diluted 40x just before use, and real-time PCR was run using 4 µL of diluted cDNA corresponding to 0.4 ng of cDNA in a total reaction volume of 10 µL. The reaction mixture was composed of 1 µL of the specific miRCURY miRNA Assay primer set (Qiagen, Hilden, Germany) and 5 µL of Fast EVA Green qPCR mastermix (Biotium, Fremont, CA 94538, USA). All reactions were run in duplicate, and a negative control without cDNA was added in each run. Samples were amplified on a Mastercycler^®^ ep Realplex (Eppendorf, Hamburg, Germany) using the following cycling conditions: 95 °C for 10 min, 40 cycles of 95 °C for 10 s and 60 °C for 1 min. For *mi-92a-1-5p* and *miR-30d-5p*, an annealing-extension temperature of 56 °C was applied; for *miR-200a-3p* and *miR-429*, it was of 58 °C. For every miRNA tested by real-time PCR, a standardization curve was created using a pool of cDNA from Bouin’s and FFPE samples. Standard curve was generated for three points using the following dilutions: 10*x* (0.4 ng/µL); 40*X* (0.1 ng/µL); 160*x* (0.025 ng/µL) for all the analyzed miRNAs. Standard curves were generated in duplicate for each miRNA and each pool of cDNA using 4 µL of diluted cDNA per replica. Cases with differences of Ct ≥ 0.5 cycle were repeated in triplicate.

### 4.6. Data Normalization

miRNA expression levels obtained by real-time qPCR were normalized using *let-7e-5p* and *miR-423-3p* as normalizing miRNAs, as returned by Bestkeeper software. In normalizing data, the geometric mean of *let-7e-5p* and *miR-423-3p* Cts was used as reference gene, while the mean of formalin and Bouin’s pooled samples was used as calibrator in the relative quantification method proposed by Livak et al. [31].

### 4.7. ddPCR

Four microliters of 40x diluted cDNAs was used in each ddPCR reaction for a direct comparison with real-time PCR. The reaction mixture contained 1x final of QX200^TM^ EvaGreen ddPCR Supermix (BioRad, Hercules, CA 94547, USA) and the miRCURY LNA PCR primer set at the appropriate concentration, which was set up experimentally for each miRNA investigated in the present study. A non-template control of deionized water was used instead of cDNA samples in each reaction. Droplet generation was performed in a QX200^TM^ Droplet Generator (BioRad, Hercules, CA 94547, USA). The droplets’ emulsion was transferred onto a 96-well plate (Eppendorf, Hamburg, Germany), which was foil-sealed twice at 179 °C for 3 s in a PX1 PCR Plate Sealer (BioRad, Hercules, CA 94547, USA) and PCR was run in a iCycler thermocycler (BioRad, Hercules, CA 94547 USA) as follows: 95 °C for 5 min; 40 cycles of 95 °C for 30 s and 56 °C for 1 min; signal stabilization at 4 °C for 5 min and 90 °C for 5 min, and final hold at 4 °C. The annealing/extension temperature for *miR-155-5p*, *miR-200a-3p*, and *miR-429* was 57 °C. After amplification, the fluorescence of each droplet was read in the QX200^TM^ Droplet Reader (BioRad, Hercules, CA 94547, USA). Droplet digital PCR data were analyzed using QuantaSoft^TM^ software and droplets’ count was fitted to a Poisson distribution to obtain the absolute concentration (copies/µL) of the target sequence.

### 4.8. Statistical Analyses

Data distribution was tested by Kurtosis test to establish the type of statistical tests (parametric or non-parametric). For normally distributed variables, the *t*-test for paired-data was run, while Wilcoxon signed rank test was used in case of non-normal distribution of data. Linear regression was run to establish the relationship between two variables (i.e., ddPCR and real-time PCR results). Pairwise Spearman’s rank analysis was carried out to investigate on variables’ dependence in case of non-normal data distribution.

All *p*-values are two-sided with values <0.05 regarded as statistically significant. Statistical analyses were performed with the Stata/SE 12 package (Stata, College Station, TX, USA).

## Figures and Tables

**Table 1:** Results of standardization curves of the analyzed miRNA.

Target	Fixative	Efficiency	Slope	Intercept
*let-7e-5p*	Formalin	0.94	−3.48	24.29
*let-7e-5p*	Bouin’s	0.97	−3.41	25.55
*miR-423-3p*	Formalin	0.91	−3.55	24.94
*miR-423-3p*	Bouin’s	0.99	−3.34	25.88
*miR-92a-1-5p*	Formalin	1.23	−2.87	32.95
*miR-92a-1-5p*	Bouin’s	0.84	−3.78	33.02
*miR-30d-5p*	Formalin	0.96	−3.43	24.00
*miR-30d-5p*	Bouin’s	0.85	−3.74	24.37
*miR-155-5p*	Formalin	0.82	−3.83	27.84
*miR-155-5p*	Bouin’s	1.04	−3.23	28.71
*miR-200a-3p*	Formalin	0.90	−3.58	24.34
*miR-200a-3p*	Bouin’s	0.88	−3.64	24.91
*miR-429*	Formalin	0.94	−3.47	25.56
*miR-429*	Bouin’s	0.85	−3.73	26.66

**Table 2:** Median Ct values of real-time PCR and median copies/µL of ddPCR results with variation coefficient for the analyzed miRNAs.

Target	Fixative	Median Ct	CV	Median Copies/µL	CV
*let-7e-5p*	Formalin	26.1	0.026	90.1	0.27
*let-7e-5p*	Bouin’s	26.3	0.063	57.6	0.65
*miR-423-3p*	Formalin	27.4	0.031	31.3	0.35
*miR-423-3p*	Bouin’s	27.2	0.048	28.1	0.49
*miR-92a-1-5p*	Formalin	33.4	0.034	0.48	0.88
*miR-92a-1-5p*	Bouin’s	33.4	0.044	0.36	1.06
*miR-30d-5p*	Formalin	24.6	0.026	152	0.53
*miR-30d-5p*	Bouin’s	25.2	0.052	96.3	0.65
*miR-155-5p*	Formalin	28.3	0.025	13.8	0.54
*miR-155-5p*	Bouin’s	29.4	0.040	7.5	0.67
*miR-200a-3p*	Formalin	26.28	0.045	46.8	0.78
*miR-200a-3p*	Bouin’s	26.97	0.068	32.2	1.16
*miR-429*	Formalin	26.95	0.042	27.2	0.85
*miR-429*	Bouin’s	27.86	0.066	14.8	1.41

**Table 3:** miRNA guanine-cytosine (GC) content and results on pairwise Spearman’s rank and regression analyses by comparing RT-qPCR and RT-ddPCR of formalin- and Bouin’s-fixed samples.

Target	GC (%)	RT-qPCR				RT-ddPCR			
		rho ^1^	*p*	R^2^	*p*	rho	*p*	R^2^	*p*
*let-7e-5p*	41	0.07	0.8	0.04	0.4	0.2	0.4	0.03	0.5
*miR-423-3p*	65	0.7	0.03	0.30	0.03	0.2	0.5	0.09	0.3
*miR-92a-1-5p*	52	0.8	0.0002	0.68	0.0003	0.7	0.007	0.84	< 0.0001
*miR-30d-5p*	50	0.7	0.01	0.38	0.02	0.7	0.005	0.64	0.0006
*miR-155-5p*	38	0.3	0.4	0.006	0.8	0.5	0.1	0.28	0.05
*miR-200a-3p*	41	0.7	0.005	0.52	0.004	0.8	0.008	0.75	0.0001
*miR-429*	36	0.7	0.004	0.46	0.008	0.6	0.01	0.65	0.0005

^1^ Spearman’s rho.

**Table 4:** Results on regression analyses comparing RT-qPCR and RT-ddPCR in paired formalin- and Bouin’s-fixed tissues.

Target	Formalin		Bouin’s	
	R^2^	*p*	R^2^	*p*
*let-7e-5p*	0.18	0.1	0.46	0.005
*miR-423-3p*	0.24	0.06	0.91	<0.00001
*miR-92a-1-5p*	0.44	0.01	0.73	0.0002
*miR-30d-5p*	0.94	<0.0001	0.99	<0.0001
*miR-155-5p*	0.007	0.8	0.90	<0.0001
*miR-200a-3p*	0.98	<0.0001	0.96	<0.0001
*miR-429*	0.95	<0.0001	0.97	<0.0001

**Table 5:** Results on RT-qPCR data normalization in paired formalin- and Bouin’s-fixed tissues.

Target	Formalin	Bouin’s	
	Median Ratio	Median Ratio	*p*
*miR-92a-1-5p*	2.7	3.9	0.1
*miR-30d-5p*	2.2	1.7	0.06
*miR-155-5p*	2.7	2.0	0.08
*miR-200a-3p*	1.1	0.8	0.3
*miR-429*	1.4	0.9	0.1

**Figure A6:**
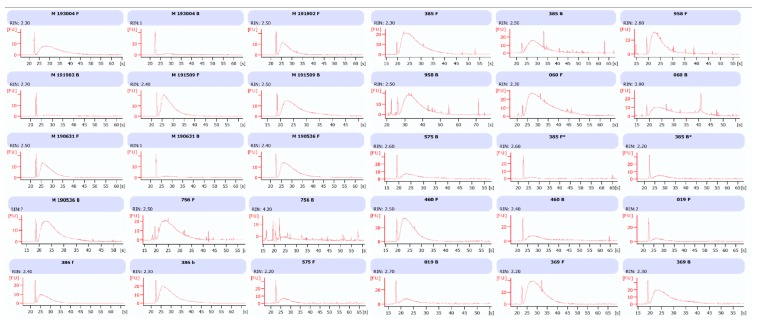
Samples are identified with the histology number or with their last three numbers, such as for 385F and 385B which refers to 190385, 958F and 958B for 188958, 060F and 060B for 188060, 756F and 756B for 188756, 386F and 386B for 128386, 575F and 575B for 117575, 385F* and 385B* for 186385, 460F and 460B for 181460, 019F and 019B for 179019, 369F and 369B for 178369. F stands for formalin and B for Bouin’s.

## Abbreviations

FFPE	Formalin-fixed and paraffin-embedded tissues
A260/280	Ratio of absorbance at 260 and 280 nm
A260/230	Ratio of absorbance at 260 and 230 nm
HGSOC	High grade serous ovarian cancer
LNA	Locked nucleic acid
miRNA	Micro RNA
RT-qPCR	Reverse transcription-quantitative polymerase chain reaction
ddPCR	Droplet digital Polymerase chain reaction

## Appendix A

This section includes additional figures.

## Appendix B

This section includes additional data related to the RIN Agilent Bioanalyzer outputs.

## References

[1] Tanca A., Addis M.F., Simula M.P., Pagnozzi D., Biosa G., Pisanu S., Garziera M., Cannizzaro R., Canzonieri V., De Re V. (2012). Evaluation of the suitability of archival Bouin-fixed paraffin-embedded tissue specimens to proteomic investigation. Electrophoresis.

[2] Ortiz-Hidalgo C. (1992). Pol Andre Bouin, MD (1870–1962). Bouin’s fixative and other contributions to medicine. Arch. Pathol. Lab. Med..

[3] Bonin S., Petrera F., Rosai J., Stanta G. (2005). DNA and RNA obtained from Bouin’s fixed tissues. J. Clin. Pathol..

[4] Muciaccia B., Vico C., Aromatario M., Fazi F., Cecchi R. (2015). Molecular analysis of different classes of RNA molecules from formalin-fixed paraffin-embedded autoptic tissues: A pilot study. Int. J. Legal Med..

[5] Saikumar J., Ramachandran K., Vaidya V.S. (2014). Noninvasive micromarkers. Clin. Chem..

[6] Peiro-Chova L., Pena-Chilet M., Lopez-Guerrero J.A., Garcia-Gimenez J.L., Alonso-Yuste E., Burgues O., Lluch A., Ferrer-Lozano J., Ribas G. (2013). High stability of microRNAs in tissue samples of compromised quality. Virchows Arch..

[7] Sanchez I., Betsou F., Culot B., Frasquilho S., McKay S.C., Pericleous S., Smith C., Thomas G., Mathieson W. (2018). RNA and microRNA Stability in PAXgene-Fixed Paraffin-Embedded Tissue Blocks After Seven Years’ Storage. Am. J. Clin. Pathol..

[8] HERCULES-Comprehensive Characterization and Effective Combinatorial Targeting of High-Grade Serous Ovarian Cancer via Single-Cell Analysis. http://www.project-hercules.eu/.

[9] Li M., Guan X., Sun Y., Mi J., Shu X., Liu F., Li C. (2014). miR-92a family and their target genes in tumorigenesis and metastasis. Exp. Cell Res..

[10] Petrillo M., Zannoni G.F., Beltrame L., Martinelli E., DiFeo A., Paracchini L., Craparotta I., Mannarino L., Vizzielli G., Scambia G. (2016). Identification of high-grade serous ovarian cancer miRNA species associated with survival and drug response in patients receiving neoadjuvant chemotherapy: A retrospective longitudinal analysis using matched tumor biopsies. Ann. Oncol..

[11] Shi M., Mu Y., Zhang H., Liu M., Wan J., Qin X., Li C. (2018). MicroRNA-200 and microRNA-30 family as prognostic molecular signatures in ovarian cancer: A meta-analysis. Medicine (Baltimore).

[12] Chen S.F., Liu Z., Chaurasiya S., Dellinger T.H., Lu J., Wu X., Qin H., Wang J., Fong Y., Yuan Y.C. (2018). Identification of core aberrantly expressed microRNAs in serous ovarian carcinoma. Oncotarget.

[13] Srinivasan M., Sedmak D., Jewell S. (2002). Effect of fixatives and tissue processing on the content and integrity of nucleic acids. Am. J. Pathol..

[14] Adam A.M.A., Refat M.S., Saad H.A., Hegab M.S. (2015). An environmentally friendly method to remove and utilize the highly toxic strychnine in other products based on proton-transfer complexation. J. Mol. Struct..

[15] Chen L., Li Y., Fu Y., Peng J., Mo M.H., Stamatakos M., Teal C.B., Brem R.F., Stojadinovic A., Grinkemeyer M. (2013). Role of deregulated microRNAs in breast cancer progression using FFPE tissue. PLoS ONE.

[16] Hoefig K.P., Thorns C., Roehle A., Kaehler C., Wesche K.O., Repsilber D., Branke B., Thiere M., Feller A.C., Merz H. (2008). Unlocking pathology archives for microRNA-profiling. Anticancer Res..

[17] Li J., Smyth P., Flavin R., Cahill S., Denning K., Aherne S., Guenther S.M., O’Leary J.J., Sheils O. (2007). Comparison of miRNA expression patterns using total RNA extracted from matched samples of formalin-fixed paraffin-embedded (FFPE) cells and snap frozen cells. BMC Biotechnol..

[18] Xi Y., Nakajima G., Gavin E., Morris C.G., Kudo K., Hayashi K., Ju J. (2007). Systematic analysis of microRNA expression of RNA extracted from fresh frozen and formalin-fixed paraffin-embedded samples. RNA.

[19] Fizpatrick M. Bouin’s Fluid Fixation. https://theolb.readthedocs.io/en/latest/histology/bouins-fluid-fixation.html.

[20] Benerini Gatta L., Cadei M., Balzarini P., Castriciano S., Paroni R., Verzeletti A., Cortellini V., De Ferrari F., Grigolato P. (2012). Application of alternative fixatives to formalin in diagnostic pathology. Eur. J. Histochem..

[21] Guillou L., Coindre J., Gallagher G., Terrier P., Gebhard S., de Saint Aubain Somerhausen N., Michels J., Jundt G., Vince D.R., Collin F. (2001). Detection of the synovial sarcoma translocation t(X;18) (SYT;SSX) in paraffin-embedded tissues using reverse transcriptase-polymerase chain reaction: A reliable and powerful diagnostic tool for pathologists. A molecular analysis of 221 mesenchymal tumors fixed in different fixatives. Hum. Pathol..

[22] Howat W.J., Wilson B.A. (2014). Tissue fixation and the effect of molecular fixatives on downstream staining procedures. Methods.

[23] Gloghini A., Canal B., Klein U., Dal Maso L., Perin T., Dalla-Favera R., Carbone A. (2004). RT-PCR analysis of RNA extracted from Bouin-fixed and paraffin-embedded lymphoid tissues. J. Mol. Diagn..

[24] Kakimoto Y., Tanaka M., Kamiguchi H., Ochiai E., Osawa M. (2016). MicroRNA Stability in FFPE Tissue Samples: Dependence on GC Content. PLoS ONE.

[25] Masuda N., Ohnishi T., Kawamoto S., Monden M., Okubo K. (1999). Analysis of chemical modification of RNA from formalin-fixed samples and optimization of molecular biology applications for such samples. Nucleic Acids Res..

[26] Baker J.R. (1958). Principles of Biological Microtechnique: A Study of Fixation and Dyeing.

[27] Racki N., Dreo T., Gutierrez-Aguirre I., Blejec A., Ravnikar M. (2014). Reverse transcriptase droplet digital PCR shows high resilience to PCR inhibitors from plant, soil and water samples. Plant Methods.

[28] Dietrich D., Uhl B., Sailer V., Holmes E.E., Jung M., Meller S., Kristiansen G. (2013). Improved PCR performance using template DNA from formalin-fixed and paraffin-embedded tissues by overcoming PCR inhibition. PLoS ONE.

[29] Hedman J., Radstrom P. (2013). Overcoming inhibition in real-time diagnostic PCR. Methods Mol. Biol..

[30] Bignotti E., Calza S., Tassi R.A., Zanotti L., Bandiera E., Sartori E., Odicino F.E., Ravaggi A., Todeschini P., Romani C. (2016). Identification of stably expressed reference small non-coding RNAs for microRNA quantification in high-grade serous ovarian carcinoma tissues. J. Cell. Mol. Med..

[31] Livak K.J., Schmittgen T.D. (2001). Analysis of relative gene expression data using real-time quantitative PCR and the 2(-Delta Delta C(T)) Method. Methods.

